# Implementing active surveillance for tuberculosis: The experiences of healthcare workers at four sites in two provinces in South Africa

**DOI:** 10.4102/safp.v64i1.5514

**Published:** 2022-07-26

**Authors:** Febisola I. Ajudua, Robert J. Mash

**Affiliations:** 1 Department of Family Medicine and Primary Care, Faculty of Medicine and Health Sciences, Stellenbosch University, Cape Town, South Africa; 2 Department of Family Medicine and Primary Care, Faculty of Health Sciences, Walter Sisulu University, Gqeberha, South Africa

**Keywords:** tuberculosis, active case finding, community-oriented primary care, community health worker (CHW), community-based services, active surveillance

## Abstract

**Background:**

The high burden of tuberculosis (TB) in South Africa (SA) is associated with uncontrolled transmission in communities and delayed diagnosis of active cases. Active surveillance for TB is provided by community-based services (CBS). Research is required to understand key factors influencing TB screening services in the CBS. This study explored the implementation of active surveillance for TB where community-oriented primary care (COPC) had been successfully implemented to identify these factors.

**Methods:**

This was a qualitative study of four established COPC sites across two provinces in SA where active surveillance for TB is implemented. Semi-structured interviews were conducted with purposively selected healthcare workers in the CBS and citizens in these communities. The recorded interviews were transcribed for data analysis using ATLAS.ti software.

**Results:**

The factors influencing active surveillance for TB were directly related to the major players in the delivery of CBS. These factors interacted in a complex network influencing implementation of active surveillance for TB. Building effective relationships across stakeholder platforms by community health workers (CHWs) was directly influenced by the training, capacity building afforded these CHWs by the district health services; and acceptability of CBS. Each factor interplayed with others to influence active surveillance for TB.

**Conclusion:**

Community health workers were central to the success of active surveillance for TB. The complex interactions of the social determinants of health and TB transmission in communities required CHWs to develop trusting relationships that responded to these issues that have impact on TB disease and linked clients to healthcare.

## Introduction

The global tuberculosis (TB) report reveals a persistent gap between the number of patients identified with active TB and the actual incidence in communities; this is further compounded by the coronavirus disease 2019 (COVID-19) pandemic.^1^ According to this report, despite three decades of efforts to fight the global TB epidemic, 10 million people were living with TB in 2020, but only 5.8 million were diagnosed. In South Africa (SA), TB remains a leading cause of morbidity and mortality, with an incidence of at least 500 per 100 000 population.^2^ One of the main problems is the delay in the diagnosis of TB,^3^ with many cases remaining hidden until very advanced.^4^ This problem is especially prevalent in poorer communities, where social determinants of health continue to drive transmission.^5^ The National TB Programme strives for early identification of active TB in at-risk groups.^4^ The main focus of the drive for improving early diagnosis is bringing TB services closer to the at-risk population through community-based TB services.^6^

South Africa has adopted a community-oriented primary care (COPC) approach to strengthen primary health care (PHC). It has been described as a continuous process by which PHC is provided to a defined community on the basis of its assessed health needs, by the planned integration of primary care practice and public health.^7^ Community-oriented primary care includes integrated facility-based and community-based health services with a focus on disease prevention, health promotion and early screening initiatives for human immunodeficiency virus (HIV), TB and more recently COVID-19.^8^ Overcoming the TB epidemic will require a multifaceted approach with effective systems for identifying active TB cases in the community.^9^ The COPC approach provides opportunities for addressing the health needs of communities, including the early diagnosis of active TB.^10^

Community-oriented primary care in the South African context relies on teams of community health workers (CHWs), supervised and supported by a nurse, who are responsible for designated groups of households.^8^ TB services are identified as essential in the pool of services provided by CHWs as part of COPC.^10^ These teams, funded for the most part by the Department of Health (DoH) but in some instances by non-governmental organisations (NGOs), provide a range of TB services.^11^ Active surveillance for TB includes all activities of healthcare workers in the community directed at early identification of suspected TB disease.^12^ The implementation of active surveillance for TB had been operational at pilot sites even before the National Department of Health (NDoH) adopted an active surveillance policy for TB. These sites provided the department with data for the development of COPC and the policy framework on CHW teams that included early identification of active TB in communities.^8^

Active surveillance involves active screening in the community, aimed at early detection of disease processes.^13^ Active surveillance for TB in high burden settings can reduce delays in diagnosis,^14^ reduce transmission^15^ and is ‘a pillar’ of the National TB Programme.^16^ Nevertheless, there are some dissenting viewpoints that argue that it does not ultimately improve treatment outcomes.^14^ Other alternative approaches have been adopted to identify TB patients, such as screening by mobile X-ray units or contact tracing.^17,18^ Active surveillance for TB is sometimes perceived as expensive and possibly unable to provide value for money.^19,20^ This perception is possibly a result of challenges experienced in implementing COPC and additional costs involved in employing and equipping CHW teams that only focus on TB surveillance.^19^ However, research shows the cost-effectiveness of CHWs when they work in a generalist and comprehensive approach.^16^

The implementation of active surveillance for TB is influenced by factors such as availability of resources, staff experience and motivation as well as collaboration between stakeholders.^21^ The effect of these factors varies between different settings.^21^ Despite the policy commitment to active TB surveillance, there are gaps in our knowledge of how to implement it at-scale in SA. The COPC model, including active surveillance for TB, has been implemented more deliberately in a number of sites throughout SA. This study aimed to explore the implementation of active surveillance for TB in four of these COPC pilot sites in SA. This study aimed to identify key lessons at these sites that could be transferred to other districts in SA, particularly in the Eastern Cape where the principal researcher practises in a context with a high burden of TB.

## Methods

### Study design

This was a qualitative study of four COPC pilot sites, two in Gauteng and two in the Western Cape province. We used semi-structured interviews to explore the experiences of various stakeholders at each site on the implementation of active surveillance for TB.

### Setting

The four sites were selected because they were well-known role models for implementation of COPC and included active surveillance for TB in the scope of practice of CHWs. The CHW teams at the sites were made up of six CHWs and an outreach team leader (OTL). The distribution of work per team was based on the municipal ward system. Each CHW was responsible for 250 households. In Gauteng, two sites were explored: one in Soweto, Johannesburg, and the other in the Tshwane District. In the Soweto site, the service was developed around the Chiawelo community practice.^4^ The CHWs provided community-based services (CBS) through home visits and were actively involved in the development of networks in the community to improve health awareness. At the Tshwane site, the service involved the use of health posts (physical structures in the community, sometimes small, prefabricated units) run by CHW teams who also provided services in the community through home visits and community campaigns. These CHWs collaborated with other stakeholders in providing health services and improving health awareness. The CHW teams in Gauteng are directly employed by the DoH and offer community-based health services. The COPC initiative in Tshwane is spearheaded by the Department of Family Medicine at University of Pretoria and is well described in the literature.^10^ The Chiawelo Community Practice in Soweto is connected to local public sector clinics and has also been described in the literature.^22^

In the Western Cape, two learning sites were explored in Cape Town: at Bishop Lavis and Nomzamo. These were pilot sites for implementation of COPC and CHWs at these sites were employed by NGOs via a contract with the DoH. In Bishop Lavis, the CHWs serve communities on the Cape Flats. In Nomzamo, the CHWs serve mostly informal settlements around the town of Somerset West around two clinics, one of which was closed because of community unrest during the week the researcher interviewed participants at this site. This however did not affect the CHWs included in the study. Their work included home-based services, inclusive of screening for health risks and community campaigns for improved health awareness. The primary care facilities provided support (via nurse practitioners or medical doctors) to the CHW teams in managing health problems identified in the community.

### Study participants

The researchers purposively selected a variety of healthcare workers involved with implementing TB surveillance in COPC and community members to explore different experiences and perspectives of the screening service. At each site one doctor, one nurse, CHWs, patients, a community leader and a manager of the programme were invited to participate. Nurses were selected because they supervised the CHW teams and were referred to as OTLs. The number of doctors and OTLs were limited at each site and all were interviewed. Sufficient interviews were conducted to ensure saturation of data from each site.

### Data collection

Invitations and arrangements for the semi-structured interviews with individual participants were made in the Soweto and Tshwane areas in October 2018. Similarly, semi-structured interviews were conducted in the Nomzamo and Bishop Lavis areas in July 2019. An interview guide was used in all interviews. Questions were based on findings from an earlier study looking at views expressed by managers of TB programmes in the resource-limited setting of the Eastern Cape province.^23^ The researchers sought to understand the views of interviewees on active surveillance for TB in COPC and more specifically on factors that influenced implementation of active surveillance for TB in the community. All interviews were conducted in English by the principal researcher. An interpreter was used if a community member preferred to converse in their home language. Interviews were audio-recorded and transcribed verbatim with the help of a professional transcriber.

The interviews in Soweto were held at the community practice. The interviews in Tshwane were held at a health post, a primary care clinic and homes of community members. In the Western Cape, interviews were held at the meeting site of the CHWs in Nomzamo, whilst the community leader was interviewed in his office. At Bishop Lavis, all parties chose to be interviewed at the primary care facility, which was considered most secure.

### Data analysis

Transcripts were checked and corrected against the audio recordings. Analysis proceeded with the use of Atlas.ti software version 8 and thematic analysis according to the framework method. The following steps were followed:

- Familiarisation: This included listening to audio recordings and reading through transcripts to become immersed in the data.
- Coding index: The researchers developed an index of codes from step 1 and organised them into categories.
- Coding: The transcribed data were then coded using the coding index.
- Charting: Similarly coded data were collated in reports based on the categories from the coding index.
- Interpretation: The reports from step 4 were interpreted to identify themes and subthemes. The range of experiences and perspectives within these themes were interpreted and any relationships between themes.

F.I.A. performed the analysis under the supervision of R.M. who gave input into the coding index and interpretation of data. In order to improve credibility, authors triangulated responses from the different interviewees and across sites. Both researchers are trained in the use of ATLAS.ti software and had experience conducting qualitative research. Altogether, 35 people were interviewed, and the overall saturation of themes was thought to have been obtained.

F.I.A. is a family physician in the Eastern Cape province who has a desire to improve active surveillance for TB in that context. She works mostly at the district hospital level and had no prior relationship with any of the interviewees.

## Findings

The profile of 35 interviewees across four sites is presented in Table 1. Altogether, this included 11 CHWs, nine community members, five OTLs, four community leaders, three family physicians and three others (two coordinators of COPC and one facility manager). Around 8–10 key informants were interviewed at each site. The findings of this study are presented as four themes and 13 subthemes as shown in Table 2.

**TABLE 1:** Profile of key informants.

Site	Family physicians	Community members	Community leaders	Outreach team leaders	Community health workers	Others	Total
Soweto	1	2	1	1	3	0	8
Tshwane	1	2	0	2	3	2	10
Nomzamo	0	3	1	1	3	0	8
Bishop Lavis	1	2	2	1	2	1	9

**Total**	**3**	**9**	**4**	**5**	**11**	**3**	**35**

**TABLE 2:** List of themes and subthemes.

Themes	Subthemes
Delivery of services for early diagnosis of active tuberculosis (TB) in the community	Approach to active TB surveillance
	Use of contact tracing
Factors related to the community	Acceptability of community-based services
	Socio-economic issues in the community
Factors related to the community health worker (CHW) teams	Building effective relationships
	Community engagement
	Multisectoral collaboration
	Information systems
Factors related to the primary health care (PHC) facility and health services	Training and capacity building of CHWs
	Support from the PHC facility
	Availability of resources and support
	Referral systems
	Monitoring and evaluation

### Delivery of services for early diagnosis of active tuberculosis in the community

#### Approach to active tuberculosis surveillance

At both sites in Gauteng, the CHW teams performed household visits and assessments, where they offered screening services (inclusive of active surveillance for TB), basic health promotion and disease prevention. If the CHWs identified people with health needs, such as TB symptoms, they booked appointments for them at the community practice or referred them to the PHC facility. In Tshwane, the CHW teams had a collaborative relationship with other NGOs working in the community, such as the Community Oriented Substance Use Program (COSUP). These organisations sometimes also provided TB services, including active surveillance. These collaborations also provided additional resources such as personnel, household assessment kits and transport.

All CHWs routinely visited households and screened for new cases of TB every few weeks to months. The frequency of repeat active surveillance visits varied across sites, influenced by workload and the burden of disease in the community. Some CHWs collected sputum in the community if the person had TB symptoms and submitted via the OTL for investigation, whilst others referred them to the PHC facility. There was no clear preference expressed between collecting sputa on site and referring patients to the facility for TB investigation; however, both had associated challenges. On the one hand, providing results to clients at follow-up visits was associated with the difficulty of finding clients who had no fixed address (such as the homeless) or moved between homes for socio-economic reasons (such as backyard dwellers). On the other hand, a CHW in Soweto described how collecting sputum during the home visit made it easier to investigate the client as this was not dependent on them attending the PHC facility. Ultimately, the CHWs ensured that patients were linked to facilities where they could access TB investigation or treatment services.

The services at Nomzamo and Bishop Lavis were similar. The CHW teams were led by professional nurses who coordinated the CBS and were employed by NGOs. The teams worked with the facility-based staff and their scope of practice spanned maternal, adult and child health, as well as health promotion and preventive care. They screened for non-communicable diseases and common infectious diseases, including TB:

‘We do outreaches and then we also go door to door and when the clinic also give us assessments to do, then we will go into that person’s house, ask that person questions and, but if I also, like I said – when I see somebody was coughing in the community when I walk past them, I will ask them and then the next morning I will go and I will give that person a referral.’ (Interview 31, CHW, Bishop Lavis)

Active surveillance for TB also occurred in the primary care facilities, in patients presenting for unrelated health problems. However, in the sites where CHWs performed active surveillance, the numbers of patients identified in the community usually exceeded the numbers identified in the facilities. Overall, patients were identified earlier through surveillance, than if they had attended the facility on their own with symptoms of TB:

‘We do a TB screen on all our patients that enter the facility. So we are currently using the integrated clinical stationery which is the new form of clinical record keeping for the Western Cape for primary care.’ (Interview 33, Professional Nurse, Bishop Lavis)

Health promotion and disease prevention were central to the CBS activities at these sites. Community health workers sometimes conducted health awareness campaigns for TB in parts of the community, especially when the incidence of TB was notably high. The CHWs, whilst conducting home visits, observed the circumstances within the home and what could be done to improve the family’s health status. These home visits served as opportunities for health education to prevent transmission of TB. For example, they might discuss issues such as opening windows for improved ventilation, letting in sunlight or visiting the clinic if they were a close contact of a known TB patient:

‘We give them a health talk about TB, HIV and AIDS, we give them health talk. Pregnancy women, we give them, we’re screening.’ (Interview 15, CHW, Tshwane)

#### Use of contact tracing for tuberculosis

In all sites, every patient identified with active TB at the facility was referred to the CHW teams to have contacts traced. However, CHWs sometimes encountered resistance from members of the community who were reluctant to have CHWs in their homes. Reasons varied from fear of stigma in the community to not accepting the CHWs as bona fide health service providers. Contact tracing was difficult in patients with no fixed address, such as homeless people or backyard dwellers:

‘We go and educate that since they were staying with this person with TB, and then he was not on treatment then, they need to come and test, because TB is contagious.’ (Interview 1, CHW, Soweto)

‘Reception is well from some of the community members, I won’t lie, besides that particular household, that guy said he does not want any person wearing navy and white [*the CHW’s uniform*], otherwise he will kill someone.’ (Interview 19, CHW, Nomzamo)

The CHWs reported that they also encountered challenges with contact tracing in the workplace, with some stating that they simply avoided this because of the difficulties associated with entering the workplace and the reluctance of people to provide the relevant information for fear of being made unemployed. Employed people did not welcome CHWs visiting them in their workplaces.

In some instances, the patients had moved across district or provincial boundaries and could not be found for contact tracing. The CHW teams often used their own networks to find patients who were difficult to trace. Community health workers stated that the TB room in the PHC facility is essential in coordinating with the CBS to provide TB services in the community.

### Factors related to the community

#### Acceptability of community-based services

Community health workers at each site spoke about improved acceptance of their services when they were formally introduced to the community, a process in most instances facilitated by recognised community leaders. This created an awareness of their services and the CHWs themselves:

‘We introduce them to the community. Say to the community these are the people that are going to help you – your health in the community – protect them.’ (Interview 32, Community leader, Nomzamo)

An example of CHWs not being accepted came from hostels within the Tshwane District. Community health workers described struggling to provide services because of the prevailing beliefs in traditional medicines and were sometimes denied entry. It was particularly difficult when the community leader in the hostel was opposed to the practice of Western medicine. On the other hand, when CHWs decided on industrial action for better working conditions at the Soweto site, community leaders supported them in their stand for better wages and encouraged them to continue working as they provided a valuable service. Some CHWs said provision of identity tags and uniforms for ease of identification also improved acceptance of their services in the community.

#### Socio-economic issues in communities

Access to services was influenced by the lack of formal housing. Community health workers described difficulties in providing ongoing services to the homeless, people in informal dwellings and backyard dwellers. These community members were very mobile, often because of the need for employment:

‘Sometimes when we want to trace defaulters, we follow the address which the patient registered with, but when we go to that area, that person is unknown.’ (Interview 14, CHW, Tshwane)

Poverty was another key factor, as patients who screened positive in the community sometimes had financial difficulties in attending the primary care facility for investigation. In addition, substance misuse and gang membership were prevalent in these communities and contributed to poor health-seeking behaviour:

‘We found lots of young men that were smoking “dagga” [*Cannabis*] on the corner and we, like I said, I can see there is something wrong with them. Their eyes, and then I asked them how long have you been coughing and they would say No, Sister, I’m fine.’ (Interview 30, CHW, Bishop Lavis)

In Tshwane, CHWs expressed difficulty in connecting with employed people who were not at home during the CHWs’ working hours.

Security was a prevailing issue in the work of CHWs and their ability to enter communities. Community leaders could be instrumental by emphasising that the community was responsible to ensure the safety of the CHWs. Community health workers described how the bags of equipment they carried made them the target of criminals. However, there were instances when CHWs found themselves protected by community members who felt responsible for them because of the work they did:

‘And I was shocked and I said, thank you caring for me. He said no, because you are working for our people in our community.’ (Interview 30, CHW, Bishop Lavis)

### Factors related to the community health worker teams

#### Building effective relationships

A people-centred approach was required for CHWs to respect patients’ beliefs and concerns. This encouraged the development of positive relationships. How CHWs asked about TB symptoms during the home visit influenced their rapport with the client. Sometimes, clients became fatigued by the long list of screening questions put to them by the CHWs. This sometimes undermined the reliability of the answers given. The CHWs’ attempts to be comprehensive could translate into the prioritisation of data collection processes over fostering relationships with their clients.

The household structure sometimes made it difficult to have private and confidential discussions with clients. Community health workers needed motivation to find ways of overcoming this challenge. In one instance, a community member feared the CHW would divulge personal information to his family during informal interactions in the community. The CHW in this instance referred the patient to the facility:

‘Just like me, I am [a] patient of HIV but they don’t know and I was very afraid to just talk about it because I was in front of my family. So I couldn’t just burst it, they don’t know. So I, I wanted a space of like this brother, I need a space of me and him one-on-one session, so that I can tell him my problem, that’s whereby I will feel comfortable.’ (Interview 13, Community member, Tshwane)

By addressing the psychosocial needs of the client, CHWs might get commitment to address other issues. For example, one CHW strengthened the relationship with the client by accessing social worker services to assist the patient in obtaining a social grant. They were then more amenable to have the grandchildren tested for TB.

The CHWs also worked to build relationships with other cadres of healthcare workers at the primary care facility who provided support for their work in the community. They considered these relationships an important resource in TB surveillance.

#### Community engagement

Community engagement helped communities understand the work of the CHW teams. It also afforded the CHWs an understanding of the prevailing issues in the community, as not all CHWs lived in the communities they served. Community meetings provided a platform for CHWs to engage with community members on health problems and services. Some even took this one step further and provided feedback to the community members on health matters and the general state of wellness in the community:

‘Those community meetings, they do help. If you have somebody representing the Department of Health, it’s very important and it brings us together with the whole community.’ (Interview 1, CHW, Soweto)

Community health workers and OTLs reported that HIV and TB were stigmatised in communities and people would deny having a problem. They addressed this challenge by providing information during health talks at community meetings.

#### Multisectoral collaboration

Community health workers recognised that the underlying factors in the community directly affected the incidence of TB. During a home visit in Tshwane, the CHW was informed of the death of a TB patient; the family was preparing for the funeral and showed this CHW the empty grocery cupboards. She wanted them to attend the clinic to test other members of the household for TB, but they were more concerned about the lack of food and organising the funeral. In responding to the complex problem of this household, she engaged with the social worker to organise food parcels. In Nomzamo, another CHW attempted to refer children for TB investigation, but the grandmother responded that she needed to first get birth certificates for them. Her priority was assistance from the social worker to get birth certificates to access the child support grant. The priorities of the family and those of the CHW did not always align; as a result, the CHW engaged stakeholders from outside the health sector to address the family’s needs for social security and gain their buy-in to respond to the issue of TB. Community health workers described being very pragmatic in addressing the challenges; they engaged the help of other stakeholders such as environmental health officers, social workers, community leaders, multidisciplinary team (MDT) from the facility and community members:

‘And in the process of our clients, sometimes, they need food. Some of our clients, they don’t have enough food to eat. Then you observe the clients, you observe. If it is something like that, we used to go to our social worker to ask for her parcels.’ (Interview 11, CHW, Tshwane)

Stakeholders might also provide support for CHW services; for example, one of the NGOs in Tshwane that was involved with substance abuse provided the CHWs with a health post. In community campaigns, the NGOs sometimes provided transport and other government departments, such as Social Development, might participate:

‘And also we do our outreach programs, we call out SASSA [*South African Social Security Agency*], we call Social Development, we call all other stakeholders to be involved so that they can talk and tell the people what they are doing.’ (Interview 27, OTL, Nomzamo)

#### Information systems

The information systems were expected to capture accurate data and provide easily interpreted reports. Accurate data could give a good overview of the performance of the CHWs. A number of CHWs were convinced that having feedback on their performance would help motivate them further. Data were collated monthly by the OTLs and submitted to the facility and to the coordinator of COPC at these sites. The electronic devices in Tshwane for capturing data made it easy to analyse and provide a report:

‘So, at the current moment I’m busy trying to make sure that all of them have got gadgets so that I can help them with interpretation of data.’ (Interview 18, Doctor, Tshwane)

However, in the same district one CHW interviewed did not have the device and spent more time writing everything by hand. The electronic data and software on the device helped CHWs to follow up clients, provide ongoing care and coordinate referrals. The lack of electronic devices made it more tedious to keep accurate records of clients seen and the numbers referred to the facilities for further care.

### Factors related to the facility and the district health services

#### Training and capacity building of community health workers

Community health workers had to undergo initial training for their work in the community. In-service training continued as an ongoing exercise to build capacity and address challenges they encountered in the course of their work. The CHWs believed this was essential in helping them understand their roles in the community, develop confidence and achieve the objectives of CBS. It was essential to address communication skills of the CHWs and ensure that CHWs asked the TB screening questions in a way likely to elicit a valid response. They also expressed the belief that the quality of their service in the community was directly affected by the opportunities afforded to them for career development:

‘And WBOT [*ward-based outreach teams*] teach us many things. We went many courses for WBOT. So I learn many things in my ward.’ (Interview 15, CHW, Tshwane)

The CHW teams were further supported by multidisciplinary professionals in the affiliated PHC facility. These professionals provided feedback to the CHW teams about their referrals, answered questions on issues raised by their clients or advised on how to manage patients in the community. This support was strengthened further during MDT meetings (when the facility and community teams came together) whilst discussing cases:

‘We are thankful for the feedback that they give us from the clinic because we are trying very hard.’ (Interview 31, CHW, Bishop Lavis)

#### Supervision of community health workers

The OTLs sometimes provided supervision during the home visit and thus improved the quality of the service according to the CHWs. Outreach team leaders assisted with solving more complex problems that went beyond the scope of the CHWs and through their input increased the confidence of clients in the ability of CHWs to help them. Community health workers also resorted to checking websites for information and thought that an Internet-based knowledge hub for CHWs would be a good idea:

‘They must know what to do when they are there. So our job is to, sometimes, we go out with them. It’s called a supervised visit to see what are they doing, what we tell them to do, are they doing it. So that’s what we do.’ (Interview 12, OTL, Tshwane)

The OTLs assisted with coordination of referrals to the facility. One respondent at Nomzamo was convinced that having a dedicated liaison person at the facility to receive referrals from the community made it easier to link people to care. Furthermore, the MDT in the facility supported a more comprehensive approach to problems in the community. The availability of the MDT strengthened the quality of the service and assisted with resolving complex problems.

#### Availability of resources and support

Active surveillance for TB was dependent on the availability of essential resources. At some of these sites, government and non-government stakeholders strengthened the CHW teams by making resources available for CBS. However, there were also challenges described with insufficient resources, such as transport and the availability of electronic devices to capture data and record their activities. Community health workers identified the availability of screening tools as a key factor; this included stationery for screening and referral to the primary care facility, and sometimes the sputum bottles in a cooler bag:

‘Some of them they don’t want to come here. But if I’ve got that bottle and that sachet to tell them, it’s fine if you don’t have the time to go you can cough inside then I close that bottle. I came with it here.’ (Interview 3, CHW, Soweto)

In Tshwane, the availability of electronic devices improved record keeping but was not available to all.

It was better to have CHWs who were resident in the community as this improved acceptability of their services and reduced the need for additional resources such as transport, which was expensive:

‘We walk, ward base(sic) is all about walking, because each CHW has to work within her catchment area, within the place that she is staying.’ (Interview 11, CHW, Tshwane)

In Tshwane, a COPC coordinator supervised the OTLs and was based in the subdistrict offices of the DoH. They assisted with monitoring and evaluation of the CBS.

#### Referral systems

The CHWs referred patients who screened positive for TB to the facility via the OTLs. The OTLs ensured that each referral was appropriate. Often referrals failed because of a lack of motivation to seek help because of other competing interests, such as going to work, fear of negative staff attitudes, lack of money for transport and expectations of long waiting times in the facility.

The CHWs were often pragmatic in addressing these difficulties. A CHW described attending MDT meetings at the facility with a list of problem cases to discuss so that they could address the problems in the community. Another described physically escorting patients to the primary care facilities to prevent them from defaulting appointments. A number of others mentioned referring to a named individual within the facility who could ensure patients’ problems are addressed promptly:

‘There’s a lot of clinical support, and then they’ve also got their CBS coordinator where they’ve got regular meetings to discuss any of their challenges.’ (Interview 28, Doctor, Bishop Lavis)

When patients did not attend the PHC facility and were not at their listed addresses, CHWs described sending the known details of the patient through a network of other CHWs to find the patient, possibly in another community. In Tshwane, the CHWs had developed a system of identifying places where the homeless often stayed during the day and visited these spaces regularly to find clients defaulting appointments.

#### Monitoring and evaluation

Monitoring and evaluation was easier if electronic information was available from every CHW involved in COPC. However, electronic devices, such as cell phones, contributed to the security problems encountered by CHWs in the community. The CHWs with no access to electronic devices used paper-based information systems. Outreach team leaders depended on information from the CHWs for providing reports. The reports from CHW teams were used in meetings with coordinators of COPC, at the facility, subdistrict and district levels to check implementation of the services:

‘Every work that we do in the community we write it down in our diaries and also the household forms are helping us because we know which house that we went to, who did we interview.’ (Interview 30, CHW, Bishop Lavis)

Evaluating the overall implementation of TB surveillance was difficult in the face of multiple service providers, running similar but parallel programmes, and each having a separate reporting system. The teams identified that integrating all data from COPC is the first step in improving coordination of services in the community:

‘So we have that problem that we have a lot of different stakeholders who are doing the same thing. So there’s also a lot of overlap. And sometimes people are not even sharing the information.’ (Interview 18, Doctor, Tshwane)

The findings highlight the successes already achieved in the implementation of active surveillance for TB in the four sites as well as issues that still need attention.

## Discussion

**FIGURE 1:**
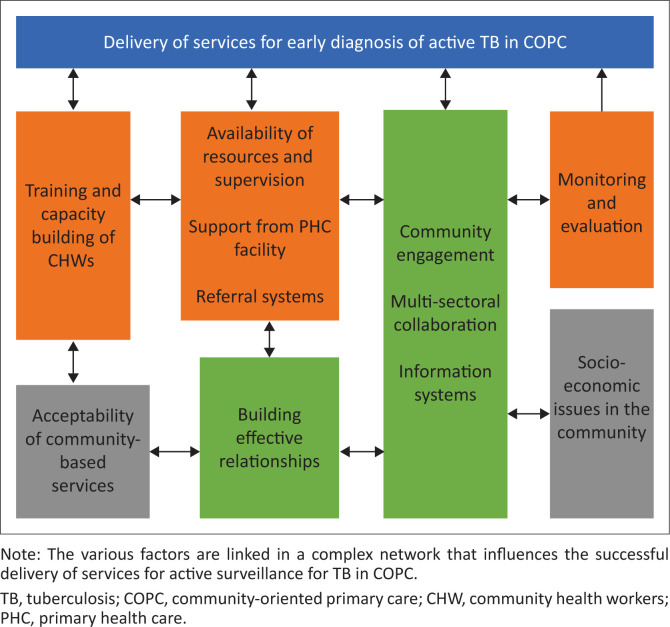
Conceptual framework of key findings.

The CHW teams were central to the success of active surveillance for TB and many of the issues raised are generic to the functioning of these teams and not to TB surveillance per se.^24^ In addition, even though the subject of this study is TB surveillance, it is hard to ignore the need for a comprehensive approach to the health needs of communities. South Africa has a quadruple burden of disease, and the TB epidemic cannot be considered in isolation, hence the need for a generalist CHW who can tackle health problems in the community. Figure 1 summarises the key findings.

### Factors related to primary care facilities and health services

The successes of the CHW teams stemmed not only from their initial training but also from the facility-based healthcare workers who developed their capacity to solve problems through ongoing training.^25^ The well-developed support systems provided by these healthcare workers strengthened the CHWs’ ability and motivation to perform their tasks. Research affirms that strong support is essential for CHWs to achieve their potential.^26^ The collaboration between facility and CBS was reflected in well-functioning referral systems. Community health workers strengthened their relationships with clients when they referred patients who received adequate help at the facility.

Inadequate record keeping made it difficult to monitor and evaluate active surveillance for TB. Tuberculosis surveillance is evaluated through reliable information systems, highlighting the need for feasible and accurate record keeping.^27^ Ongoing monitoring and evaluation by the OTLs and coordinators allowed CHWs to review their work and helped to identify gaps in the services.^28^ This was more feasible when CHWs were equipped with electronic devices to record activities. Data could then be analysed and supervisors provide feedback.^29^ A feedback system, when it was available at these sites, strengthened the CHWs’ understanding of their contributions to the healthcare system, including their contribution to the TB programme. A study reporting on the use of electronic devices by CHW teams reflected on how the analysis of electronic data, collected via an app, provided information to target interventions.^30^ Paper-based records were more difficult to keep accurately and even more difficult to analyse in order to provide feedback to the CHWs. This finding was further affirmed in another study evaluating household assessment data in Cape Town.^31^ Although CHWs reported that smartphones may make them the targets for crime, evidence is accumulating on the use of mobile devices in the delivery of CBS to accurately record activities, easily analyse data and help target interventions.^32^

### Factors related to the community health worker teams

The findings showed how CHW teams supported all three key levers of effective PHC from their contribution to primary care, to multisectoral collaboration and engaging with the communities they served.^33^ In terms of primary care, implementing active surveillance for TB relied on the motivation of CHWs to ask screening questions, interpret the answers, link those screening positive to care or other services and overcome any challenges in completing this process.

In terms of multisectoral collaboration, the CHWs’ work in the community in this study relied on their capacity to form relationships with the primary care facility as well as other government departments and stakeholders.^26^ The CHWs recognised that their services did not end just with the task of conducting a home visit or assisting with a health campaign, but required them to solve complex health and social problems in households and communities. Even when CHW programmes are designed as vertical systems specific to active surveillance for TB, implementation requires the CHWs to respond to complex issues in households that go beyond the focus on TB.^34^ To respond adequately to these issues, CHWs described how they had to draw on resources from the wider PHC team and other stakeholders in the community they serve.^34^

Although active surveillance is often conceptualised as a simple case-finding service for TB, the reality is that complex social needs and issues often take priority in households; this was observed across all sites in this study.^35^ If CHWs cannot help with these issues, they may not be able to motivate individuals to access TB services. Assisting with these social issues, such as assuring food security or obtaining social grants, requires multisectoral collaboration. The CHWs could interact with NGOs, social workers, the Department of Social Development and known persons in their network that could assist, but often found they were unable to address all the problems. The World Health Organization (WHO) identified that a structured approach for multisectoral interventions is required to respond adequately to the complex problems often encountered in communities.^28^ Other research affirmed the need for policymakers to strengthen the approach to these complex issues through improved collaboration between government departments at higher levels of governance in order to commit the required resources and develop a multisectoral response.^25^ Multisectoral collaboration should be enabled at micro-, meso- and macro-levels of the health system.

Community engagement is recognised to be an effective means of improving health and health-seeking behaviour in communities.^36^ Living and working in their own communities enhanced the motivation of CHWs and increased their sense of responsibility to make a difference. The CHWs devised various strategies to engage communities, such as forming relationships with recognised leaders, engaging with groups of people with common interests and talking to community members about TB and how to access healthcare.

### Factors related to the community

The CHWs’ success was dependent to some extent on how acceptable their services were to the community.^37^ Community leaders could facilitate acceptance and cooperation of community members with TB surveillance and linkage to care. Research has affirmed that community support also enhances the motivation and performance of CHWs.^38^ The community that accepted their CHWs was more likely to ensure their safety and protect them especially when the CHW is known to the community. The persistent problem of security was addressed by engaging communities through their leaders to make them more responsible.^36^ However, in communities with major socio-economic problems, such as high levels of unemployment, substance abuse and high rates of petty crime, security remained a problem. It is in instances such as this that multisectoral collaboration may assist with addressing these complex problems and groups such as the police and neighbourhood watch may also assist CHWs to be safe. Recommendation 14 of the WHO policy guideline for system support of CHWs is about mobilising community health resources to identify these complex and often context-specific problems and implementing interventions with community participation.^39^ A scoping review of CBS in SA also identified similar security challenges in CBS.^40^

### Limitations

It is important to highlight that the sites included in this study were learning sites for the development of COPC in SA.^22,30^ The services at these sites were developed over time and have been the subject of a number of studies on COPC. The CHWs at these sites were quite experienced and seemed very motivated to address problems encountered in the course of their daily work in active surveillance for TB. It is also important to note that these sites were urban. The findings, therefore, are not easily generalised to rural areas and districts where COPC is less developed. However, the findings point towards learning from best practice, which was the intention of this study, and can assist with the development of TB surveillance in other parts of the country.

### Recommendations

Facility-based members of PHC should ensure that they provide training and build the capacity of CHWs to solve the problems that they encounter in the community. In addition, they should ensure that referral pathways and linkage to care are working so that CHW referrals are effective. District and facility management should ensure that CHWs have the resources they need to function effectively in terms of infrastructure, transport, equipment, supplies and supervision. District and facility management should ensure a functional health information system that captures the work of the CHWs, including TB surveillance, and ideally this should be electronic and Internet-based so that all captured information is immediately available for analysis and reporting. The management structures at district level should foster multisectoral task teams that can contribute to address the complex issues that often influence TB surveillance in communities. The PHC teams should help identify the priority health problems and underlying determinants to be addressed.

Community health workers should be trained in communication skills that assist them to build effective relationships with clients and form alliances with other stakeholders. Their training should also include methods of community engagement and involvement to improve health in the communities they serve. In addition, training should explore how to address complex problems through engaging the local multidisciplinary PHC team and other stakeholders. Career pathways and bursaries for CHWs’ development may increase their motivation and commitment to work.

More effort needs to be made to engage community leaders and community members to make the goal of community engagement and involvement a reality. This may improve acceptance of and trust in CHWs’ services, such as TB surveillance, and increase their safety. In addition, it may lead to opportunities to provide feedback to communities on health issues and together prioritise and address more complex underlying problems.

Further research is needed to evaluate interventions that address the issues identified and in particular look at improving multisectoral action, community engagement and empowerment. Furthermore, longitudinal research that measures outcomes of active surveillance for TB would better describe the influence of the factors described here. There is a need for effective monitoring systems that monitor and measure the various aspects of TB surveillance in the National TB Programme. These tools and systems require further research into their design and effectiveness.

The findings of this study will be incorporated into a survey of healthcare workers in the Eastern Cape province in order to quantify how important these factors are to TB surveillance in that context. In addition, the findings will inform quality improvement looking at TB surveillance in the Eastern Cape province.

## Conclusion

The CHW teams were central to the success of active surveillance for TB in the four sites visited. A number of factors related to the key stakeholders in CBS influenced implementation of TB surveillance. The well-established support structures availed these teams of training, availability of resources, effective referral systems with support from the primary care facility and equipping CHWs for effective functioning at these sites. It was evident that CHWs resourced with electronic data collection capacity captured activities that were easy to analyse and evaluate for feedback. Community health workers were often required to address complex issues in the course of delivering active surveillance for TB, highlighting the need for adequately equipping them to address these issues. The complex interactions of the social determinants of health and TB transmission in the community required CHWs who could engage with the community and develop trusting relationships to adequately respond to these complex issues by linking clients to healthcare whilst liaising with other sectors to address problems that impact TB infection in the lives of their clients and their communities. Acceptability of the CHW teams in the community was influenced in part by their ability to develop trusting relationships with clients. Further research is required to develop systems for intersectoral collaboration in addressing the socio-economic problems that often contribute to the transmission of TB in disadvantaged communities.

## References

[1] WHO. Global tuberculosis report 2021. Geneva: WHO; 2021.

[2] Statistics South Afric. Mortality and causes of death in South Africa, 2018: Findings from death notification [homepage on the Internet]. 2021 [cited 2021 Dec 3]. Available from: http://www.statssa.gov.za/publications/P03093/P030932009.pdf

[3] Getnet F, Demissie M, Assefa N, Mengistie B, Worku A. Delay in diagnosis of pulmonary tuberculosis in low-and middle-income settings: Systematic review and meta-analysis. BMC Pulm Med. 2017;17(1):202. 10.1186/s12890-017-0551-y 29237451 PMC5729407

[4] National Department of Health. Joint Review of HIV, TB and PMTCT programmes in South Africa October 2013 annexes to main report. Unpublished. 2013 (October).

[5] Carter DJ, Glaziou P, Lönnroth K, et al. The impact of social protection and poverty elimination on global tuberculosis incidence: A statistical modelling analysis of Sustainable Development Goal 1. Lancet Glob Health. 2018;6(5):E514–E522. 10.1016/S2214-109X(18)30195-5 29580761 PMC5968370

[6] Stop TB Partnership Strategic Initiative. Finding missing people with TB in communities [homepage on the Internet]. 2018 [cited 2020 Oct 12]. Available from: https://stoptb-strategicinitiative.org/elearning/wp-content/uploads/2019/04/STBFG_03.pdf

[7] Abramson J. Community-oriented primary care – Strategy, approaches, and practice: A review. Public Health Rev. 1988;16(1–2):35–98. 3073435

[8] South African National Department of Health. Policy framework and strategy for ward-based primary healthcare outreach teams 2018/19 – 2023/24 [homepage on the Internet]. 2018 [cited 2019 Dec 28] p. 16–20. Available from: https://rhap.org.za/wp-content/uploads/2018/04/Policy-WBPHCOT-4-April-2018-1.pdf

[9] Blok L, Sahu S, Creswell J, Alba S, Stevens R, Bakker MI. Comparative meta-analysis of tuberculosis contact investigation interventions in eleven high burden countries. PLoS One. 2015;10(3):e0119822. 10.1371/journal.pone.0119822 25812013 PMC4374904

[10] Kinkel H, Marcus T, Memon S, Bam N, Hugo J. Community oriented primary care in Tshwane District, South Africa: Assessing the first phase of implementation. Afr J Prim Health Care Fam Med. 2012;5(1):1–9. 10.4102/phcfm.v5i1.477

[11] Friedemann H, Marcus T, Bam N, Hugo J. Two years in: A status quo report on ward based outreach team (WBOT). In: Fourth South African TB Conference - TB management in Tshwane District. 2014 June, 10–13, Durban; 2014.

[12] Kranzer K. Intensified tuberculosis case finding among HIV-infected individuals [serial online]. Continuing Med Educ. 2011 [cited 2016 May 22];29:418. Available from: http://www.cmej.org.za/index.php/cmej/article/view/2272/2034

[13] Lönnroth K, Corbett E, Golub J, Uplekar M, Weil D, Raviglione M. State of the art systematic screening for active tuberculosis: Rationale, definitions and key considerations. Int J Tuberc Lung Dis. 2013;17:289–298. 10.5588/ijtld.12.0797 23407219

[14] Shewade HD, Gupta V, Satyanarayana S, et al. Active versus passive case finding for tuberculosis in marginalised and vulnerable populations in India: Comparison of treatment outcomes. Glob Health Action. 2019;12(1):1656451. 10.1080/16549716.2019.1656451 31475635 PMC6735288

[15] Gashu Z, Jerene D, Ensermu M, et al. The yield of community-based ‘retrospective’ tuberculosis contact investigation in a high burden setting in Ethiopia. PLoS One. 2016;11(8):e0160514. 10.1371/journal.pone.0160514 27483160 PMC4970728

[16] Daviaud E, Besada D, Budlender D, Sanders D, Kerber K. Saving lives, saving costs: Investment case for community health workers in South Africa [homepage on the Internet]. 2018 [cited 2021 Apr 10]. Available from: https://www.samrc.ac.za/sites/default/files/files/2017-10-30/SavingLivesSavingCosts.pdf

[17] Chheng P, Nsereko M, Malone LL, et al. Tuberculosis case finding in first-degree relative contacts not living with index tuberculosis cases in Kampala, Uganda. Clin Epidemiol. 2015;7:411–419. 26508888 10.2147/CLEP.S82389 PMC4610802

[18] Lorent N, Choun K, Thai S, et al. Community-based active tuberculosis case finding in poor urban settlements of Phnom Penh, Cambodia: A feasible and effective strategy. PLoS One. 2014;9(3):e92754. 10.1371/journal.pone.0092754 24675985 PMC3968028

[19] Ayles H, Muyoyeta M, Du Toit E, et al. Effect of household and community interventions on the burden of tuberculosis in southern Africa: The ZAMSTAR community-randomised trial. Lancet. 2013;382(9899):1183–1194. 10.1016/S0140-6736(13)61131-9 23915882

[20] Kranzer K, Lawn SD, Meyer-Rath G, et al. Feasibility, yield, and cost of active tuberculosis case finding linked to a mobile HIV service in Cape Town, South Africa: A cross-sectional study. PLoS Med. 2012;9(8):1–11. 10.1371/journal.pmed.1001281 PMC3413719 22879816

[21] Biermann O, Lönnroth K, Caws M, Viney K. Factors influencing active tuberculosis case-finding policy development and implementation: A scoping review. BMJ Open. 2019;9(12):1–12. 10.1136/bmjopen-2019-031284 PMC6924749 31831535

[22] Schutz E. Pioneering holistic healthcare in Soweto. Health E-news; 2020 July 31.

[23] Ajudua FI, Mash RJ. Implementing active surveillance for TB – The views of managers in a resource limited setting, South Africa. PLoS One. 2020;15(10):e0239430. 10.1371/journal.pone.0239430 33006993 PMC7531829

[24] Schneider H, Lehmann U. From community health workers to community health systems: Time to widen the horizon? Health Syst Reform. 2016;2(2):112–118. 10.1080/23288604.2016.1166307 31514640

[25] Perry HB, Hodgins S. Health for the people: Past, current, and future contributions of national community health worker programs to achieving global health goals. Glob Health Sci Pract. 2021;9(1):1–9. 10.9745/GHSP-D-20-00459 33795359 PMC8087430

[26] Kok MC, Ormel H, Broerse JEW, et al. Optimising the benefits of community health workers’ unique position between communities and the health sector: A comparative analysis of factors shaping relationships in four countries. Glob Public Health. 2017;12(11):1404–1432. 10.1080/17441692.2016.1174722 27133127

[27] Karimuribo E, Mutagahywa E, Sindato C, et al. A smartphone app (AfyaData) for innovative one health disease surveillance from community to national levels in Africa: Intervention in disease surveillance. JMIR Public Health Surveill. 2017;3(4):e94. 10.2196/publichealth.7373 29254916 PMC5748470

[28] WHO, Unicef. Operational framework for primary health care: Transforming vision into action [homepage on the Internet]. 2020 [cited 2021 July 31] p. 1–106. Available from: https://apps.who.int/iris/handle/10665/337641

[29] Feroz A, Jabeen R, Saleem S. Using mobile phones to improve community health workers performance in low-and-middle-income countries. BMC Public Health. 2020;20(1):1–6. 10.1186/s12889-020-8173-3 31931773 PMC6958627

[30] Reji E, Marcus T. Peer-learning reviews to improve Gauteng community-oriented primary care: Findings from AitaHealth^TM^ -enabled ward-based outreach teams. Afr J Prim Health Care Fam Med. 2020;12(1):a2155. 10.4102/phcfm.v12i1.2155 PMC7136793 32242431

[31] Mash R, Du Pisanie L, Swart C, Van der Merwe E. Evaluation of household assessment data collected by community health workers in Cape Town, South Africa. S Afr Fam Pract. 2020;62(1):1–6. 10.4102/safp.v62i1.5168 PMC8378136 33314942

[32] Brey Z, Mash R, Goliath C, Roman D. Home delivery of medication during coronavirus disease 2019, Cape Town, South Africa: Short report. Afr J Prim Health Care Fam Med. 2020;12(1):1–4. 10.4102/phcfm.v12i1.2449 PMC7284162 32501022

[33] Shewade HD, Govindarajan S, Thekkur P, et al. Public health action: Experience of active tuberculosis case finding in nearly 5 million households in India. Public Health Action. 2016;6(4):242–246.28123961

[34] Palazuelos D, Jabateh LM, Choi M, et al. Early lessons from launching an innovative community health household model across 3 country contexts. Glob Health Sci Pract. 2021;16:S168–S178. 10.9745/GHSP-D-20-00405 PMC7971378 33727328

[35] Palazuelos D, Farmer PE, Mukherjee J. Community health and equity of outcomes: The partners in health experience. Lancet Glob Health. 2018;6(5):e491–e493. 10.1016/S2214-109X(18)30073-1 29653618

[36] Cyril S, Smith BJ, Possamai-inesedy A, et al. Exploring the role of community engagement in improving the health of disadvantaged populations: A systematic review. Glob Health Action. 2015;8(1):29842. 10.3402/gha.v8.29842 26689460 PMC4685976

[37] Ndambo MSK, Munyaneza F, Aron M, et al. A qualitative study on the role of community health workers in influencing social connectedness using the household model: A case study of Neno District, Malawi. Durham, NC: Research Square, 2020; p. 1–21. 10.21203/rs.3.rs-81409/v1

[38] Nxumalo N, Goudge J, Manderson L. Community health workers, recipients’ experiences and constraints to care in South Africa – A pathway to trust. AIDS Care. 2016;28(Suppl 4):61–71. 10.1080/09540121.2016.1195484 27345712

[39] WHO. WHO guideline on health policy and system support to optimize community health worker programmes. Geneva: WHO, 2020; p. 1–116. 30431747

[40] Mash R, Christian C, Chigwanda R V. Alternative mechanisms for delivery of medication in South Africa: A scoping review. S Afr Fam Pract. 2021;63(1):1–8. 10.4102/safp.v63i1.5274 PMC8424755 34476963

